# Bis(2-amino-4-methyl­pyridinium) terephthalate tetra­hydrate

**DOI:** 10.1107/S1600536810025651

**Published:** 2010-07-07

**Authors:** Madhukar Hemamalini, Hoong-Kun Fun

**Affiliations:** a X-ray Crystallography Unit, School of Physics, Universiti Sains Malaysia, 11800 USM, Penang, Malaysia

## Abstract

In the crystal structure of the title salt, 2C_6_H_9_N_2_^+^·C_8_H_4_O_4_^2−^·4H_2_O, the terephthalate carboxyl­ate groups inter­acts with the 2-amino-4-methyl­pyridinium cations *via* a pair of N—H⋯O hydrogen bonds, forming an *R*_2_^2^(8) ring motif. The water mol­ecules form an *R*_6_^6^(12) ring motif through O—H⋯O hydrogen bonds and these motifs are fused, forming a supra­molecular chain along the *c* axis. The *R*_2_^2^(8) and *R*_6_^6^(12) ring motifs are connected *via* O—H⋯O hydrogen bonds. In addition, π–π stacking inter­actions are observed between the pyridinium rings [centroid–centroid distance = 3.522 (12) Å].

## Related literature

For details of non-covalent inter­actions, see: Desiraju (2007 ▶); Corna *et al.* (2004 ▶); Aakeröy & Seddon (1993 ▶). For background to the chemistry of substituted pyridines, see: Pozharski *et al.* (1997 ▶); Katritzky *et al.* (1996 ▶). For the applications of terephthalic acid, see: Serre *et al.* (2007 ▶); Mukherjee *et al.* (2004 ▶); Sun *et al.* (2000 ▶); Lynch & Jones (2004 ▶); Spencer *et al.* (2004 ▶); Devi & Muthiah (2007 ▶). For details of hydrogen bonding, see: Jeffrey & Saenger (1991 ▶); Jeffrey (1997 ▶); Scheiner (1997 ▶). For hydrogen-bond motifs, see: Bernstein *et al.* (1995 ▶). For bond-length data, see: Allen *et al.* (1987 ▶). For the stability of the temperature controller used in the data collection, see: Cosier & Glazer (1986 ▶).
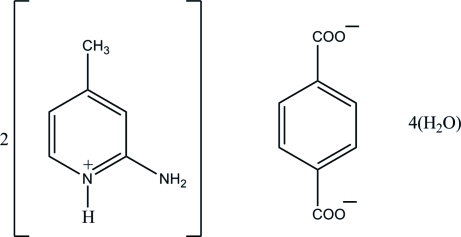


## Experimental

### 

#### Crystal data

2C_6_H_9_N_2_^+^·C_8_H_4_O_4_^2−^·4H_2_O
*M* *_r_* = 454.48
Monoclinic, 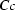

*a* = 17.6290 (16) Å
*b* = 13.8091 (13) Å
*c* = 9.2518 (9) Å
β = 93.940 (2)°
*V* = 2246.9 (4) Å^3^
*Z* = 4
Mo *K*α radiation
μ = 0.11 mm^−1^
*T* = 100 K
0.45 × 0.27 × 0.07 mm

#### Data collection

Bruker APEXII DUO CCD area-detector diffractometer
Absorption correction: multi-scan (*SADABS*; Bruker, 2009 ▶) *T*_min_ = 0.955, *T*_max_ = 0.992
12651 measured reflections
3275 independent reflections
3004 reflections with *I* > 2σ(*I*)
*R*_int_ = 0.031

#### Refinement

*R*[*F* ^2^ > 2σ(*F* ^2^)] = 0.036
*wR*(*F* ^2^) = 0.101
*S* = 1.06
3275 reflections
347 parameters
2 restraints
H atoms treated by a mixture of independent and constrained refinement
Δρ_max_ = 0.46 e Å^−3^
Δρ_min_ = −0.20 e Å^−3^

### 

Data collection: *APEX2* (Bruker, 2009 ▶); cell refinement: *SAINT* (Bruker, 2009 ▶); data reduction: *SAINT*; program(s) used to solve structure: *SHELXTL* (Sheldrick, 2008 ▶); program(s) used to refine structure: *SHELXTL*; molecular graphics: *SHELXTL*; software used to prepare material for publication: *SHELXTL* and *PLATON* (Spek, 2009 ▶).

## Supplementary Material

Crystal structure: contains datablocks global, I. DOI: 10.1107/S1600536810025651/ci5123sup1.cif

Structure factors: contains datablocks I. DOI: 10.1107/S1600536810025651/ci5123Isup2.hkl

Additional supplementary materials:  crystallographic information; 3D view; checkCIF report

## Figures and Tables

**Table 1:** Hydrogen-bond geometry (Å, °)

*D*—H⋯*A*	*D*—H	H⋯*A*	*D*⋯*A*	*D*—H⋯*A*
N1*A*—H1*NA*⋯O2	0.95 (3)	1.75 (3)	2.698 (2)	172 (3)
N2*A*—H2*NA*⋯O1	0.83 (3)	2.10 (3)	2.906 (2)	165 (3)
N2*A*—H3*NA*⋯O1^i^	0.91 (3)	1.97 (3)	2.866 (2)	171 (2)
N1*B*—H1*NB*⋯O4^ii^	1.02 (5)	1.72 (5)	2.723 (2)	169 (3)
N2*B*—H2*NB*⋯O3^ii^	0.90 (4)	1.88 (4)	2.765 (2)	170 (3)
N2*B*—H3*NB*⋯O3*W*^iii^	0.93 (3)	1.97 (3)	2.894 (2)	174 (3)
O1*W*—H1*W*1⋯O3*W*	0.76 (4)	2.03 (3)	2.781 (2)	174 (4)
O1*W*—H2*W*1⋯O4*W*	0.89 (4)	1.91 (4)	2.779 (2)	167 (3)
O2*W*—H1*W*2⋯O4^iv^	0.88 (3)	1.86 (3)	2.741 (2)	175 (3)
O2*W*—H2*W*2⋯O4*W*	0.88 (3)	1.90 (3)	2.772 (2)	176 (3)
O3*W*—H1*W*3⋯O3^v^	0.77 (4)	1.95 (4)	2.721 (2)	178 (5)
O3*W*—H2*W*3⋯O1*W*^vi^	0.86 (4)	1.89 (3)	2.742 (2)	168 (3)
O4*W*—H1*W*4⋯O2	0.92 (4)	1.81 (4)	2.689 (2)	161 (3)
O4*W*—H2*W*4⋯O2*W*^vi^	0.80 (3)	1.93 (3)	2.716 (2)	171 (3)

Symmetry codes: (i) 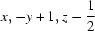; (ii) 
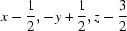
; (iii) 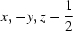; (iv) 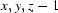; (v) 
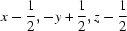
; (vi) 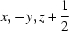.

**Fig. 1.**
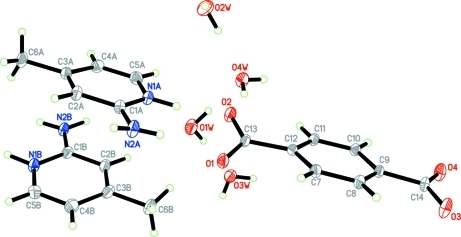
The asymmetric unit of the title compound. Displacement ellipsoids are drawn at the 50% probability level.

**Fig. 2.**
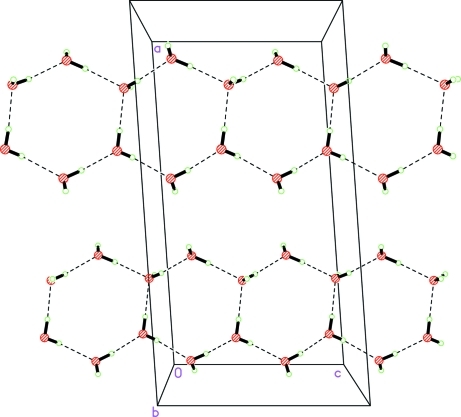
View of the supramolecular chain made up of water molecules along the c-axis.

**Fig. 3.**
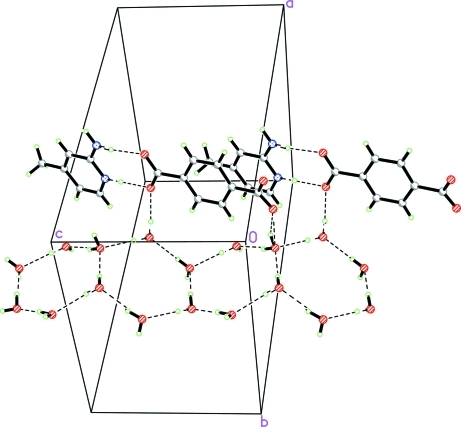
Hydrogen bonding pattern in the title compound.

## supplementary crystallographic information

### Comment

Supramolecular architectures assembled *via* various delicate noncovalent interactions such as hydrogen bonds, π–π stacking and electrostatic interactions, etc., have attracted intense interest in recent years because of their fascinating structural diversity and potential applications for functional materials (Desiraju, 2007; Corna *et al.*, 2004). Especially, the application of intermolecular hydrogen bonds is a well known and efficient tool in the field of organic crystal design owing to its strength and directional properties (Aakeröy & Seddon, 1993). Pyridine and its derivatives play an important role in heterocyclic chemistry (Pozharski *et al.*, 1997; Katritzky *et al.*, 1996). They are often involved in hydrogen-bond interactions (Jeffrey & Saenger, 1991; Jeffrey, 1997; Scheiner, 1997). Terephthalic acid (H_2_TPA), a rod-like aromatic diacid, has often been used in the synthesis of metal-organic frameworks as a linker molecule (Serre *et al.*, 2007; Mukherjee *et al.*, 2004; Sun *et al.*, 2000).  Recently, with the increase in interest in controlling the crystalline structures of organic-based solid-state materials, H_2_TPA is being increasingly employed in constructing supramolecular structures (Lynch & Jones, 2004; Spencer *et al.*, 2004; Devi & Muthiah, 2007). Since our aim is to study some interesting hydrogen-bonding interactions, the crystal structure of the title compound is presented here.

The asymmetric unit of the title salt contains two 2-amino-4-methylpyridinium cations (A and B), one terephthalate anion and four water molecules (Fig. 1). Each 2-amino-4-methylpyridinium cation is planar, with a maximum deviation of 0.008 (2) Å for atom C2A (molecule A) and 0.005 (2) Å for atom C3B (molecule B). The protonation of atoms N1A and N1B lead to a slight increase in  C1A—N1A—C5A [122.10 (16)°] and C1B—N1B—C5B [122.09 (16)°] angles. The bond lengths (Allen *et al.*, 1987) and angles are normal.

In the crystal structure, the terephthalate carboxylate groups interacts with 2-amino-4-methylpyridinium cations *via* a pair of N—H···O hydrogen bonds, forming an *R*_2_^2^(8) ring motif (Bernstein *et al.*, 1995). An *R*_6_^6^(12) ring motif is formed by water molecules through O—H···O (Table 1) hydrogen bonds and these motifs fuse to form a one-dimensional supramolecular chain along the *c*-axis (Fig. 2). Further, the *R*_2_^2^(8) and *R*_6_^6^(12) motifs are connected *via* O—H···O hydrogen bonds (Fig. 3). The crystal structure is further stabilized by π–π  interactions between N1A/C1A–C5A and N1B/C1B–C5B pyridinium rings at (x, y, z) [centroid-to-centroid distance = 3.522 (1) Å].

### Experimental

A hot methanol solution (20 ml) of 2-amino-4-methylpyridine (54 mg, Aldrich) and terephthalic acid (83 mg, Merck) were mixed and warmed over a heating magnetic stirrer hotplate for a few minutes. The resulting solution was allowed to cool slowly at room temperature and crystals of the title compound appeared after a few days.

### Refinement

O- and N-bound H atoms were located from a difference Fourier map and were refined freely [N–H= 0.83 (3)–1.01 (5) Å and O–H = 0.75 (3)–0.92 (3) Å]. The remaining hydrogen atoms were positioned geometrically [C–H = 0.93 or 0.96 Å] and were refined using a riding model, with *U*_iso_(H) = 1.2 or 1.5 *U*_eq_(C).  A rotating group model was used for the methyl groups. In the absence of significant anomalous dispersion, 2680 Friedel pairs were merged before the final refinement.

### Crystal data

**Table:**

2C_6_H_9_N_2_^+^·C_8_H_4_O_4_^2^^−^·4H_2_O	*F*(000) = 968
*M**_r_* = 454.48	*D*_x_ = 1.343 Mg m^−^^3^
Monoclinic, *C**c*	Mo *K*α radiation, λ = 0.71073 Å
Hall symbol: C -2yc	Cell parameters from 4430 reflections
*a* = 17.6290 (16) Å	θ = 2.3–30.0°
*b* = 13.8091 (13) Å	µ = 0.11 mm^−^^1^
*c* = 9.2518 (9) Å	*T* = 100 K
β = 93.940 (2)°	Plate, colourless
*V* = 2246.9 (4) Å^3^	0.45 × 0.27 × 0.07 mm
*Z* = 4	

### Data collection

**Table:**

Bruker APEXII DUO CCD area-detector diffractometer	3275 independent reflections
Radiation source: fine-focus sealed tube	3004 reflections with *I* > 2σ(*I*)
graphite	*R*_int_ = 0.031
φ and ω scans	θ_max_ = 30.1°, θ_min_ = 1.9°
Absorption correction: multi-scan (*SADABS*; Bruker, 2009)	*h* = −21→24
*T*_min_ = 0.955, *T*_max_ = 0.992	*k* = −19→13
12651 measured reflections	*l* = −13→13

### Refinement

**Table:**

Refinement on *F*^2^	Primary atom site location: structure-invariant direct methods
Least-squares matrix: full	Secondary atom site location: difference Fourier map
*R*[*F*^2^ > 2σ(*F*^2^)] = 0.036	Hydrogen site location: inferred from neighbouring sites
*wR*(*F*^2^) = 0.101	H atoms treated by a mixture of independent and constrained refinement
*S* = 1.06	*w* = 1/[σ^2^(*F*_o_^2^) + (0.0657*P*)^2^ + 0.2622*P*] where *P* = (*F*_o_^2^ + 2*F*_c_^2^)/3
3275 reflections	(Δ/σ)_max_ = 0.001
347 parameters	Δρ_max_ = 0.46 e Å^−^^3^
2 restraints	Δρ_min_ = −0.20 e Å^−^^3^

### Special details

**Table:**

Experimental. The crystal was placed in the cold stream of an Oxford Cryosystems Cobra open-flow nitrogen cryostat (Cosier & Glazer, 1986) operating at 100.0 (1) K.
Geometry. All s.u.'s (except the s.u. in the dihedral angle between two l.s. planes) are estimated using the full covariance matrix. The cell s.u.'s are taken into account individually in the estimation of s.u.'s in distances, angles and torsion angles; correlations between s.u.'s in cell parameters are only used when they are defined by crystal symmetry. An approximate (isotropic) treatment of cell s.u.'s is used for estimating s.u.'s involving l.s. planes.
Refinement. Refinement of F^2^ against ALL reflections. The weighted R-factor wR and goodness of fit S are based on F^2^, conventional R-factors R are based on F, with F set to zero for negative F^2^. The threshold expression of F^2^ > 2σ(F^2^) is used only for calculating R-factors(gt) etc. and is not relevant to the choice of reflections for refinement. R-factors based on F^2^ are statistically about twice as large as those based on F, and R- factors based on ALL data will be even larger.

### Fractional atomic coordinates and isotropic or equivalent isotropic displacement parameters (Å^2^)

**Table:**

	*x*	*y*	*z*	*U*_iso_*/*U*_eq_	
N1A	0.73749 (9)	0.27051 (12)	0.16796 (17)	0.0163 (3)	
N2A	0.75478 (10)	0.43694 (12)	0.17137 (18)	0.0204 (3)	
C1A	0.73275 (10)	0.35697 (14)	0.09938 (19)	0.0163 (4)	
C2A	0.70424 (11)	0.35868 (15)	−0.0481 (2)	0.0187 (4)	
H2AA	0.7016	0.4170	−0.0983	0.022*	
C3A	0.68056 (10)	0.27471 (14)	−0.11685 (19)	0.0173 (4)	
C4A	0.68604 (11)	0.18620 (14)	−0.0396 (2)	0.0188 (4)	
H4AA	0.6702	0.1286	−0.0841	0.023*	
C5A	0.71489 (11)	0.18657 (15)	0.1009 (2)	0.0202 (4)	
H5AA	0.7192	0.1285	0.1518	0.024*	
C6A	0.64919 (12)	0.27652 (16)	−0.2720 (2)	0.0230 (4)	
H6AA	0.6584	0.3389	−0.3133	0.034*	
H6AB	0.5954	0.2645	−0.2762	0.034*	
H6AC	0.6736	0.2274	−0.3257	0.034*	
N1B	0.48583 (10)	0.31843 (13)	−0.04463 (18)	0.0195 (3)	
N2B	0.49724 (10)	0.15247 (12)	−0.02353 (17)	0.0184 (3)	
C1B	0.50869 (11)	0.23962 (14)	0.03428 (19)	0.0169 (4)	
C2B	0.54266 (11)	0.25447 (16)	0.1759 (2)	0.0183 (4)	
H2BA	0.5582	0.2015	0.2326	0.022*	
C3B	0.55264 (11)	0.34599 (15)	0.2296 (2)	0.0200 (4)	
C4B	0.52752 (12)	0.42658 (16)	0.1435 (2)	0.0230 (4)	
H4BA	0.5336	0.4894	0.1788	0.028*	
C5B	0.49433 (12)	0.41005 (15)	0.0082 (2)	0.0220 (4)	
H5BA	0.4773	0.4622	−0.0487	0.026*	
C6B	0.58818 (13)	0.36063 (18)	0.3806 (2)	0.0271 (5)	
H6BA	0.6173	0.3043	0.4099	0.041*	
H6BB	0.5490	0.3707	0.4461	0.041*	
H6BC	0.6210	0.4162	0.3822	0.041*	
O1	0.79418 (8)	0.41120 (10)	0.47912 (14)	0.0196 (3)	
O2	0.80222 (9)	0.25260 (10)	0.43950 (15)	0.0196 (3)	
O3	0.95157 (10)	0.35431 (12)	1.18466 (16)	0.0258 (3)	
O4	0.93553 (9)	0.19471 (11)	1.17111 (15)	0.0227 (3)	
C7	0.87740 (10)	0.39144 (13)	0.75006 (19)	0.0156 (3)	
H7A	0.8791	0.4520	0.7063	0.019*	
C8	0.90527 (10)	0.37998 (14)	0.89289 (19)	0.0158 (3)	
H8A	0.9248	0.4331	0.9447	0.019*	
C9	0.90425 (10)	0.28948 (14)	0.95947 (18)	0.0148 (3)	
C10	0.87640 (10)	0.20985 (14)	0.87956 (19)	0.0153 (3)	
H10A	0.8771	0.1488	0.9219	0.018*	
C11	0.84752 (11)	0.22144 (13)	0.73637 (19)	0.0156 (3)	
H11A	0.8286	0.1681	0.6840	0.019*	
C12	0.84683 (10)	0.31250 (13)	0.67150 (18)	0.0138 (3)	
C13	0.81214 (10)	0.32704 (14)	0.51865 (18)	0.0147 (3)	
C14	0.93282 (10)	0.27846 (14)	1.11620 (19)	0.0165 (4)	
O1W	0.63068 (10)	0.06139 (12)	0.38459 (17)	0.0271 (3)	
O2W	0.85423 (10)	0.02585 (12)	0.18024 (18)	0.0268 (3)	
O3W	0.56061 (10)	0.01269 (12)	0.63491 (16)	0.0242 (3)	
O4W	0.78771 (9)	0.05874 (11)	0.43863 (16)	0.0224 (3)	
H1NA	0.7560 (17)	0.265 (2)	0.267 (3)	0.024 (7)*	
H2NA	0.7743 (15)	0.432 (2)	0.255 (3)	0.022 (6)*	
H3NA	0.7636 (14)	0.489 (2)	0.115 (3)	0.019 (6)*	
H1NB	0.463 (3)	0.307 (3)	−0.147 (5)	0.077 (14)*	
H2NB	0.4765 (19)	0.150 (2)	−0.115 (4)	0.038 (8)*	
H3NB	0.5148 (17)	0.100 (2)	0.032 (3)	0.033 (8)*	
H1W1	0.6143 (17)	0.050 (2)	0.456 (4)	0.025 (7)*	
H2W1	0.680 (2)	0.051 (2)	0.404 (4)	0.040 (9)*	
H1W2	0.8800 (16)	0.080 (2)	0.172 (3)	0.025 (7)*	
H2W2	0.8348 (16)	0.035 (2)	0.264 (3)	0.025 (7)*	
H1W3	0.529 (2)	0.049 (3)	0.649 (4)	0.059 (12)*	
H2W3	0.5807 (17)	−0.003 (2)	0.719 (4)	0.034 (8)*	
H1W4	0.7949 (18)	0.123 (3)	0.461 (3)	0.035 (8)*	
H2W4	0.8036 (18)	0.029 (2)	0.508 (4)	0.031 (7)*	

### Atomic displacement parameters (Å^2^)

**Table:**

	*U*^11^	*U*^22^	*U*^33^	*U*^12^	*U*^13^	*U*^23^
N1A	0.0186 (7)	0.0172 (7)	0.0128 (7)	−0.0016 (6)	−0.0011 (6)	0.0010 (5)
N2A	0.0271 (8)	0.0185 (7)	0.0147 (7)	−0.0044 (6)	−0.0042 (6)	0.0014 (6)
C1A	0.0166 (8)	0.0170 (8)	0.0151 (8)	−0.0016 (7)	−0.0005 (6)	0.0016 (6)
C2A	0.0203 (9)	0.0220 (9)	0.0136 (7)	−0.0013 (7)	−0.0008 (6)	0.0026 (6)
C3A	0.0146 (8)	0.0234 (9)	0.0140 (8)	0.0014 (7)	0.0007 (6)	0.0006 (7)
C4A	0.0200 (9)	0.0182 (8)	0.0182 (8)	−0.0020 (7)	0.0015 (7)	−0.0031 (7)
C5A	0.0209 (9)	0.0184 (9)	0.0215 (9)	0.0021 (7)	0.0024 (7)	0.0027 (7)
C6A	0.0234 (10)	0.0321 (11)	0.0125 (8)	−0.0007 (8)	−0.0051 (7)	−0.0014 (7)
N1B	0.0219 (8)	0.0207 (8)	0.0153 (7)	−0.0009 (6)	−0.0021 (6)	0.0001 (6)
N2B	0.0225 (8)	0.0195 (8)	0.0127 (7)	−0.0017 (6)	−0.0024 (6)	−0.0006 (6)
C1B	0.0162 (8)	0.0211 (9)	0.0135 (8)	−0.0020 (6)	0.0013 (6)	0.0009 (6)
C2B	0.0172 (8)	0.0240 (9)	0.0137 (8)	−0.0006 (7)	0.0002 (6)	0.0005 (6)
C3B	0.0174 (9)	0.0295 (10)	0.0133 (8)	−0.0029 (7)	0.0020 (6)	−0.0019 (7)
C4B	0.0250 (10)	0.0221 (9)	0.0217 (9)	−0.0004 (8)	−0.0004 (7)	−0.0039 (7)
C5B	0.0232 (9)	0.0227 (9)	0.0198 (9)	0.0027 (7)	−0.0003 (7)	−0.0001 (7)
C6B	0.0282 (11)	0.0370 (12)	0.0151 (9)	0.0003 (9)	−0.0048 (8)	−0.0064 (8)
O1	0.0276 (7)	0.0164 (6)	0.0140 (6)	0.0043 (5)	−0.0034 (5)	0.0006 (5)
O2	0.0293 (7)	0.0149 (6)	0.0138 (6)	0.0008 (5)	−0.0032 (5)	−0.0006 (5)
O3	0.0373 (8)	0.0238 (7)	0.0151 (6)	−0.0085 (6)	−0.0064 (6)	0.0005 (5)
O4	0.0309 (8)	0.0196 (7)	0.0165 (6)	−0.0048 (6)	−0.0053 (5)	0.0033 (5)
C7	0.0165 (8)	0.0154 (8)	0.0146 (8)	0.0003 (6)	−0.0007 (6)	0.0007 (6)
C8	0.0160 (8)	0.0173 (8)	0.0138 (7)	−0.0027 (6)	−0.0014 (6)	−0.0012 (6)
C9	0.0143 (8)	0.0198 (8)	0.0103 (7)	0.0005 (6)	−0.0001 (6)	0.0015 (6)
C10	0.0183 (8)	0.0152 (8)	0.0124 (8)	0.0010 (7)	0.0002 (6)	0.0017 (6)
C11	0.0180 (8)	0.0161 (8)	0.0126 (8)	0.0004 (7)	−0.0006 (6)	−0.0006 (6)
C12	0.0136 (8)	0.0166 (8)	0.0112 (7)	0.0015 (6)	0.0010 (6)	0.0009 (6)
C13	0.0163 (8)	0.0177 (8)	0.0099 (7)	−0.0002 (6)	−0.0003 (6)	0.0006 (6)
C14	0.0177 (8)	0.0203 (9)	0.0109 (7)	−0.0035 (7)	−0.0028 (6)	0.0009 (6)
O1W	0.0280 (8)	0.0343 (9)	0.0189 (7)	−0.0029 (7)	−0.0002 (6)	0.0039 (6)
O2W	0.0357 (9)	0.0214 (7)	0.0236 (7)	−0.0049 (6)	0.0044 (6)	−0.0045 (6)
O3W	0.0289 (8)	0.0237 (7)	0.0197 (7)	0.0027 (6)	0.0002 (6)	−0.0012 (5)
O4W	0.0306 (8)	0.0169 (7)	0.0194 (7)	−0.0007 (6)	−0.0007 (6)	0.0001 (5)

### Geometric parameters (Å, °)

**Table:**

N1A—C1A	1.352 (2)	C4B—H4BA	0.93
N1A—C5A	1.361 (3)	C5B—H5BA	0.93
N1A—H1NA	0.95 (3)	C6B—H6BA	0.96
N2A—C1A	1.334 (2)	C6B—H6BB	0.96
N2A—H2NA	0.83 (3)	C6B—H6BC	0.96
N2A—H3NA	0.91 (3)	O1—C13	1.253 (2)
C1A—C2A	1.421 (2)	O2—C13	1.267 (2)
C2A—C3A	1.374 (3)	O3—C14	1.256 (2)
C2A—H2AA	0.93	O4—C14	1.263 (2)
C3A—C4A	1.416 (3)	C7—C8	1.387 (2)
C3A—C6A	1.503 (2)	C7—C12	1.398 (2)
C4A—C5A	1.363 (3)	C7—H7A	0.93
C4A—H4AA	0.93	C8—C9	1.394 (3)
C5A—H5AA	0.93	C8—H8A	0.93
C6A—H6AA	0.96	C9—C10	1.395 (2)
C6A—H6AB	0.96	C9—C14	1.510 (2)
C6A—H6AC	0.96	C10—C11	1.395 (2)
N1B—C1B	1.356 (2)	C10—H10A	0.93
N1B—C5B	1.361 (3)	C11—C12	1.393 (2)
N1B—H1NB	1.01 (5)	C11—H11A	0.93
N2B—C1B	1.327 (3)	C12—C13	1.515 (2)
N2B—H2NB	0.90 (3)	O1W—H1W1	0.75 (3)
N2B—H3NB	0.93 (3)	O1W—H2W1	0.89 (3)
C1B—C2B	1.418 (2)	O2W—H1W2	0.88 (3)
C2B—C3B	1.365 (3)	O2W—H2W2	0.88 (3)
C2B—H2BA	0.93	O3W—H1W3	0.76 (5)
C3B—C4B	1.421 (3)	O3W—H2W3	0.86 (3)
C3B—C6B	1.505 (3)	O4W—H1W4	0.92 (3)
C4B—C5B	1.364 (3)	O4W—H2W4	0.79 (3)
			
C1A—N1A—C5A	122.10 (16)	C4B—C3B—C6B	120.57 (19)
C1A—N1A—H1NA	121.5 (17)	C5B—C4B—C3B	118.71 (19)
C5A—N1A—H1NA	116.4 (17)	C5B—C4B—H4BA	120.6
C1A—N2A—H2NA	118.8 (19)	C3B—C4B—H4BA	120.6
C1A—N2A—H3NA	115.1 (16)	N1B—C5B—C4B	121.01 (18)
H2NA—N2A—H3NA	122 (2)	N1B—C5B—H5BA	119.5
N2A—C1A—N1A	119.37 (16)	C4B—C5B—H5BA	119.5
N2A—C1A—C2A	122.50 (17)	C3B—C6B—H6BA	109.5
N1A—C1A—C2A	118.13 (16)	C3B—C6B—H6BB	109.5
C3A—C2A—C1A	120.45 (18)	H6BA—C6B—H6BB	109.5
C3A—C2A—H2AA	119.8	C3B—C6B—H6BC	109.5
C1A—C2A—H2AA	119.8	H6BA—C6B—H6BC	109.5
C2A—C3A—C4A	119.13 (16)	H6BB—C6B—H6BC	109.5
C2A—C3A—C6A	120.58 (18)	C8—C7—C12	120.35 (17)
C4A—C3A—C6A	120.29 (18)	C8—C7—H7A	119.8
C5A—C4A—C3A	119.09 (17)	C12—C7—H7A	119.8
C5A—C4A—H4AA	120.5	C7—C8—C9	120.59 (16)
C3A—C4A—H4AA	120.5	C7—C8—H8A	119.7
N1A—C5A—C4A	121.08 (18)	C9—C8—H8A	119.7
N1A—C5A—H5AA	119.5	C8—C9—C10	119.16 (15)
C4A—C5A—H5AA	119.5	C8—C9—C14	120.08 (15)
C3A—C6A—H6AA	109.5	C10—C9—C14	120.77 (16)
C3A—C6A—H6AB	109.5	C11—C10—C9	120.31 (16)
H6AA—C6A—H6AB	109.5	C11—C10—H10A	119.8
C3A—C6A—H6AC	109.5	C9—C10—H10A	119.8
H6AA—C6A—H6AC	109.5	C12—C11—C10	120.29 (16)
H6AB—C6A—H6AC	109.5	C12—C11—H11A	119.9
C1B—N1B—C5B	122.09 (16)	C10—C11—H11A	119.9
C1B—N1B—H1NB	118 (3)	C11—C12—C7	119.24 (15)
C5B—N1B—H1NB	120 (3)	C11—C12—C13	120.85 (15)
C1B—N2B—H2NB	117 (2)	C7—C12—C13	119.90 (16)
C1B—N2B—H3NB	116.5 (19)	O1—C13—O2	124.18 (16)
H2NB—N2B—H3NB	126 (3)	O1—C13—C12	118.25 (16)
N2B—C1B—N1B	118.66 (16)	O2—C13—C12	117.57 (16)
N2B—C1B—C2B	123.14 (18)	O3—C14—O4	124.01 (16)
N1B—C1B—C2B	118.19 (18)	O3—C14—C9	117.32 (16)
C3B—C2B—C1B	120.39 (19)	O4—C14—C9	118.66 (16)
C3B—C2B—H2BA	119.8	H1W1—O1W—H2W1	103 (3)
C1B—C2B—H2BA	119.8	H1W2—O2W—H2W2	101 (3)
C2B—C3B—C4B	119.60 (17)	H1W3—O3W—H2W3	106 (4)
C2B—C3B—C6B	119.81 (19)	H1W4—O4W—H2W4	106 (3)
			
C5A—N1A—C1A—N2A	179.05 (18)	C3B—C4B—C5B—N1B	−0.6 (3)
C5A—N1A—C1A—C2A	−1.0 (3)	C12—C7—C8—C9	0.9 (3)
N2A—C1A—C2A—C3A	−178.48 (19)	C7—C8—C9—C10	1.5 (3)
N1A—C1A—C2A—C3A	1.6 (3)	C7—C8—C9—C14	−178.37 (18)
C1A—C2A—C3A—C4A	−1.0 (3)	C8—C9—C10—C11	−2.2 (3)
C1A—C2A—C3A—C6A	178.99 (18)	C14—C9—C10—C11	177.62 (17)
C2A—C3A—C4A—C5A	−0.2 (3)	C9—C10—C11—C12	0.5 (3)
C6A—C3A—C4A—C5A	179.84 (18)	C10—C11—C12—C7	1.9 (3)
C1A—N1A—C5A—C4A	−0.2 (3)	C10—C11—C12—C13	−176.98 (17)
C3A—C4A—C5A—N1A	0.8 (3)	C8—C7—C12—C11	−2.6 (3)
C5B—N1B—C1B—N2B	178.77 (19)	C8—C7—C12—C13	176.24 (17)
C5B—N1B—C1B—C2B	−0.2 (3)	C11—C12—C13—O1	161.04 (18)
N2B—C1B—C2B—C3B	−179.74 (19)	C7—C12—C13—O1	−17.8 (3)
N1B—C1B—C2B—C3B	−0.8 (3)	C11—C12—C13—O2	−18.0 (3)
C1B—C2B—C3B—C4B	1.1 (3)	C7—C12—C13—O2	163.16 (17)
C1B—C2B—C3B—C6B	179.48 (19)	C8—C9—C14—O3	5.2 (3)
C2B—C3B—C4B—C5B	−0.4 (3)	C10—C9—C14—O3	−174.63 (18)
C6B—C3B—C4B—C5B	−178.8 (2)	C8—C9—C14—O4	−175.75 (19)
C1B—N1B—C5B—C4B	0.9 (3)	C10—C9—C14—O4	4.4 (3)

### Hydrogen-bond geometry (Å, °)

**Table:**

*D*—H···*A*	*D*—H	H···*A*	*D*···*A*	*D*—H···*A*
N1A—H1NA···O2	0.95 (3)	1.75 (3)	2.698 (2)	172 (3)
N2A—H2NA···O1	0.83 (3)	2.10 (3)	2.906 (2)	165 (3)
N2A—H3NA···O1^i^	0.91 (3)	1.97 (3)	2.866 (2)	171 (2)
N1B—H1NB···O4^ii^	1.02 (5)	1.72 (5)	2.723 (2)	169 (3)
N2B—H2NB···O3^ii^	0.90 (4)	1.88 (4)	2.765 (2)	170 (3)
N2B—H3NB···O3W^iii^	0.93 (3)	1.97 (3)	2.894 (2)	174 (3)
O1W—H1W1···O3W	0.76 (4)	2.03 (3)	2.781 (2)	174 (4)
O1W—H2W1···O4W	0.89 (4)	1.91 (4)	2.779 (2)	167 (3)
O2W—H1W2···O4^iv^	0.88 (3)	1.86 (3)	2.741 (2)	175 (3)
O2W—H2W2···O4W	0.88 (3)	1.90 (3)	2.772 (2)	176 (3)
O3W—H1W3···O3^v^	0.77 (4)	1.95 (4)	2.721 (2)	178 (5)
O3W—H2W3···O1W^vi^	0.86 (4)	1.89 (3)	2.742 (2)	168 (3)
O4W—H1W4···O2	0.92 (4)	1.81 (4)	2.689 (2)	161 (3)
O4W—H2W4···O2W^vi^	0.80 (3)	1.93 (3)	2.716 (2)	171 (3)

Symmetry codes: (i) *x*, −*y*+1, *z*−1/2; (ii) *x*−1/2, −*y*+1/2, *z*−3/2; (iii) *x*, −*y*, *z*−1/2; (iv) *x*, *y*, *z*−1; (v) *x*−1/2, −*y*+1/2, *z*−1/2; (vi) *x*, −*y*, *z*+1/2.
